# Therapeutic effects of adipose-derived mesenchymal stem cells against brain death-induced remote organ damage and post-heart transplant acute rejection

**DOI:** 10.18632/oncotarget.21433

**Published:** 2017-09-30

**Authors:** Hon-Kan Yip, Mel S. Lee, Cheuk-Kwan Sun, Kuan-Hung Chen, Han-Tan Chai, Pei-Hsun Sung, Kun-Chen Lin, Sheung-Fat Ko, Chun-Man Yuen, Chu-Feng Liu, Pei-Lin Shao, Fan-Yen Lee

**Affiliations:** ^1^ Division of Cardiology, Department of Internal Medicine, Kaohsiung Chang Gung Memorial Hospital and Chang Gung University College of Medicine, Kaohsiung, Taiwan; ^2^ Institute for Translational Research in Biomedicine, Kaohsiung Chang Gung Memorial Hospital, Kaohsiung, Taiwan; ^3^ Center for Shockwave Medicine and Tissue Engineering, Kaohsiung Chang Gung Memorial Hospital and Chang Gung University College of Medicine, Kaohsiung, Taiwan; ^4^ Department of Medical Research, China Medical University Hospital, China Medical University, Taichung, Taiwan; ^5^ Department of Nursing, Asia University, Taichung, Taiwan; ^6^ Department of Orthopedics, Kaohsiung Chang Gung Memorial Hospital and Chang Gung University College of Medicine, Kaohsiung, Taiwan; ^7^ Department of Emergency Medicine, E-Da Hospital, I-Shou University School of Medicine for International Students, Kaohsiung, Taiwan; ^8^ Department of Anesthesiology, Kaohsiung Chang Gung Memorial Hospital and Chang Gung University College of Medicine, Kaohsiung, Taiwan; ^9^ Department of Radiology, Kaohsiung Chang Gung Memorial Hospital and Chang Gung University College of Medicine, Kaohsiung, Taiwan; ^10^ Division of Neurosurgery, Department of Surgery, Kaohsiung Chang Gung Memorial Hospital and Chang Gung University College of Medicine, Kaohsiung, Taiwan; ^11^ Department of Emergency Medicine, Kaohsiung Chang Gung Memorial Hospital and Chang Gung University College of Medicine, Kaohsiung, Taiwan; ^12^ Division of Thoracic and Cardiovascular Surgery, Department of Surgery, Kaohsiung Chang Gung Memorial Hospital and Chang Gung University College of Medicine, Kaohsiung, Taiwan

**Keywords:** brain death, heart transplantation, inflammation, immunogenicity, remote organ damage

## Abstract

We tested the hypothesis that allogenic adipose-derived mesenchymal stem cells (ADMSCs) alleviated brain death (BD)-induced remote organ damage and events of post heart-transplant acute rejection. To determine the impact of BD on remote organ damage, adult-male F344 rats (n=24) were categorized sham-control (SC), BD and BD^MSC^ (allogenic ADMSC/1.2 × 10^6^ cells/derived from F344 by intravenous transfusion 3 h after BD procedure). To determine the protective effect of allogenic ADMSCs, animals (n=8/each group in F344/Lewis) were categorized into groups BD-T(F344 heart transplanted into Lewis by 6h after BD), BD-T^MSC(D1/3)^ (BD induction for 6h then heart transplantation, and allogenic ADMSCs transfusion at days 1 and 5 after heart transplantation), BD-T^MSC(3h)^ (BD + ADMSC/1.2 × 10^6^ cells at 3h and heart transplantation at 6h after BD) and BD-T^MSC(3h, D1/3)^ [BD + ADMSC/1.2 × 10^6^ cells at 3h and heart transplantation at 6h after BD, then ADMSC therapy by days 1/3]. At day 5 post procedure, liver, kidney and heart specimens showed higher molecular-cellular levels of inflammation, immune reaction, apoptosis and fibrosis in BD than in SC that were reversed in BD^MSC^ (all P < 0.0001). These molecular-cellular expressions and circulating/splenic levels of innate/adoptive immune cells were higher in BD-T, lowest in BD-T^MSC(3h, D1/3)^ and higher BD-T^MSC(3h)^ in than BD-T^MSC(D1/3)^, whereas heart function showed an opposite pattern among the four groups (all P < 0.001). In conclusion, ADMSCs suppressed BD-caused remote organ damage and heart-transplant rejection.

## INTRODUCTION

Advanced congestive heart failure (CHF) in its terminal stage is the most important contributor to cardiac death among all cardiovascular diseases [1–3]. Despite state-of-the-art pharmacomodulation [4–13] and refinement of therapeutic strategies [14–17], the incidence of death in patients with decompensated CHF remains extremely high with an estimated incidence of more than 50% per year and has remained unchanged over the last few decades [1–3]. Therefore, heart transplantation is the last life-saving resort for patients with advanced decompensated CHF [18–21]. However, two critical problems: (1) an extremely limited number of donors (i.e., shortage of donor hearts), and (2) rejection after heart transplantations remain unresolved. With regard to the first issue, to date there is no effective strategy. Intriguingly, for the second issue, although pre-transplant major histocompatibility complex (MHC) matching between donor and recipient has been routine and advanced immunosuppressive regimens have been extensively utilized after heart transplantation, rejection is still a bottleneck for transplant success. This suggests that unidentified confounders may exist.

The donors for heart transplantation are always brain-death (BD) victims. Interestingly, our previous study has shown that, as compared with normal control and risk-control subjects, patients with acute ischemic stroke [4] had significantly higher circulating level of N-terminal pro-brain natriuretic peptide (NT-proBNP) [22]. Additionally, as compared to less severe IS patients, patients with more severe IS also had significantly higher incidence of advanced CHF, myocardial infarction, and in-hospital death as well as lower left ventricular ejection fraction (LVEF) [22, 23]. Other recent experimental and clinical observation studies [24–27] have not only consistently revealed findings similar to ours [22, 23], but also exhibited up to more than 25% patients with lower LVEF even at the early stage of BD [28]. These findings [22–28] raised the hypothesis that organ function may be impaired in BD patients.

Our clinical observational study has previously shown that circulating inflammatory mediators were significantly increased in patients after acute IS [23, 29–33] and furthermore higher in those of severe IS patients [23, 29–33]. Furthermore, the link between severity of brain damage and inflammatory and immune reactions has been identified in our and other previous studies [23, 29, 32–36]. Importantly, these biomarkers have been shown to be predictive of the prognostic outcome of patients after IS in our previous studies [23, 29–33].

Experimental studies have demonstrated that vigorous inflammatory reaction and hyper-reactive immune response commonly occur in circulation [37] and in major organs such as liver, heart and kidney after BD in animals [34–36]. These major organs are consistently injured from the inflammatory and immune reactions after BD [23, 29–38]. These findings led to the hypothesis that BD commonly causes molecular-cellular perturbations and malfunction of remote organs mainly through enhancing inflammatory and immune responses, resulting in increased risk of organ transplantation failure [39].

A body of studies has shown that mesenchymal stem cells (MSCs) have the capacity to be anti-inflammatory [40–43] and immunomodulatory [44–48]. Consistently, our studies have shown that adipose-derived mesenchymal stem cells (ADMSC) possess anti-inflammatory, anti-apoptotic, anti-fibrosis, anti-oxidative stress and immunomodulation activities in various settings of ischemia-related organ damage, including acute IS [42-44, 49]. However, no data has been presented to address the therapeutic impact of MSCs against BD-induced damage in remote organs and acute rejection after heart transplantation. Thus, using an experimental model of BD and heart transplantation, we tested the hypothesis that ADMSC therapy might effectively protect remote organs from BD-induced injury and acute rejection after heart transplantation in rodents.

## RESULTS

### Pathophysiological findings, inflammatory biomarkers and apoptotic cells in circulation, and circulating/splenic immune cells at 6 h after the BD procedure

**Figure 1:**
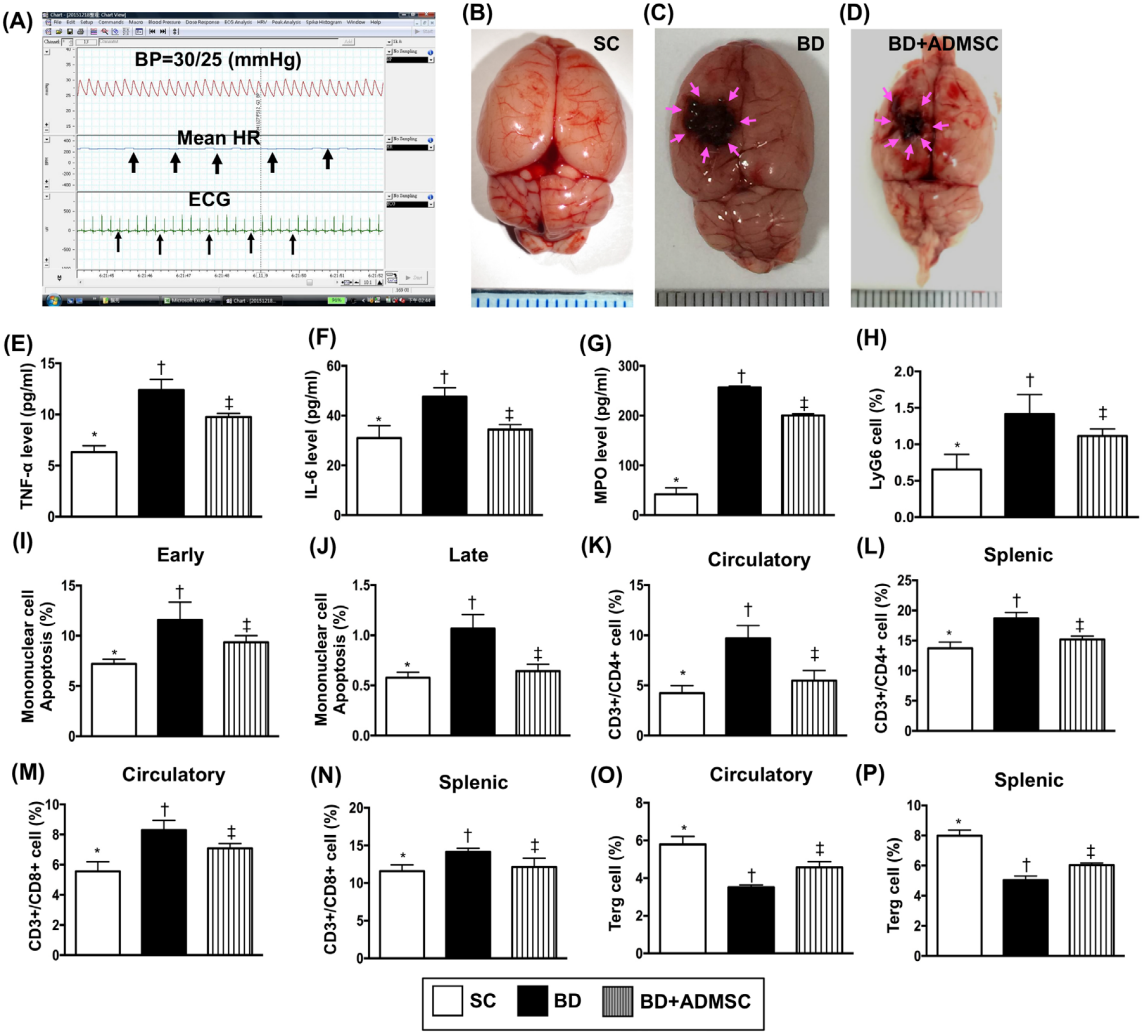
Pathophysiological findings, ELISA assessment and flow cytometric analyses of inflammatory/apoptotic biomarkers and immune cells in circulation at 6 h after the BD procedure **(A)** Illustrating the hemodynamic status (i.e., maximal mean blood pressure in all animals was recorded as 42.66 ± 3.8 mmHg/n=8), mean heart rate/255.98 ± 11.2 beat per minute (black arrows), and electrocardiogram recording (black arrows) in brain death (BD) animals. (**B** to **D**) Illustrating grossly anatomical picture of brain in sham control (B), BD animal (C) and BD animal + ADMSC treated animals **(**D**)**. The pink arrows indicated the brain damage (i.e., by pressure compression) in BD animals. **(E)** ELISA analysis of circulating level of tumor necrosis factor (TNF)-α by 6 h after BD procedure, * vs. other groups with different symbols (†, ‡), p<0.001. **(F)** ELISA analysis of circulating level of interleukin-6 by 6 h after BD procedure, * vs. other groups with different symbols (†, ‡), p<0.01. **(G)** Flow cytometric analysis of MPO level, * vs. other groups with different symbols (†, ‡), p<0.0001. **(H)** Flow cytometric analysis of LyG6 cell levels, * vs. other groups with different symbols (†, ‡), p<0.01. **(I)** Early apoptosis, * vs. other groups with different symbols (†, ‡), p<0.001. **(J)** Late apoptosis, * vs. other groups with different symbols (†, ‡), p<0.001. **(K)** Circulating levels of CD3+/CD4+ cells (helper T cells), * vs. other groups with different symbols (†, ‡), p<0.001. **(L)** Splenic levels of CD3+/CD4+ cells, * vs. other groups with different symbols (†, ‡), p<0.001. **(M)** Circulating levels of CD3+/CD8+ cells (cytotoxic T cells), * vs. other groups with different symbols (†, ‡), p<0.001. **(N)** Splenic levels of CD3+/CD8+ cells, * vs. other groups with different symbols (†, ‡), p<0.01. **(O)** Circulating levels of CD4+CD25+Foxp3+ cells (Treg cells), * vs. other bars with different symbols (†, ‡), p<0.01. **(P)** Splenic levels of Treg cells, * vs. other groups with different symbols (†, ‡), p<0.001. All statistical analyses were performed by one-way ANOVA, followed by Bonferroni multiple comparison post hoc test (n=8). Symbols (*, †, ‡) indicate significance (at 0.05 level). BD = brain death; group A1 = sham control; group A2 = brain death; group A3 = brain death + adipose-derived mesenchymal stem cell (ADMSC).

Figure 1 illustrates the pathophysiological findings (i.e., heart rate and blood pressure) in BD F344 rats. The results showed that during the BD period (maintained for 6 h), the mean heart rate was about 255.98 beats/min and the mean blood pressure was 42.6 mmHg, suggesting the experimental model of BD was successfully created. Additionally, the gross anatomical picture shows that the pressure area of the brain has the appearance of local hemorrhagic, supporting an appropriate application of pressure-induced BD in F433 rats.

To elucidate the circulating level of inflammatory reaction after BD, the enzyme-linked immunosorbent assay (ELISA) and flow cytometric analysis were utilized in the present study. The results of ELISA showed that at 6 h after BD procedure, circulating levels of tumor necrosis factor (TNF)- α, interleukin (IL)-6, and myeloperoxidase (MPO), three indicators of inflammatory markers implicating innate immune response, were higher in the BD group than in the sham control (SC) and the BD + ADMSC group (BD^MSC^), and significantly higher in BD^MSC^ group than in SC.

The flow cytometric analysis showed that the circulating level of LyG6, an indicator of inflammatory cells showed an identical pattern to the circulating level of inflammatory biomarkers among the three groups. Consistently, the circulating level of early and late apoptotic cells exhibited an identical pattern of LyG6 among the three groups.

To assess the circulating and splenic levels of immune cells, flow cytometric analysis was utilized in the present study. The results demonstrated that circulating and splenic levels CD3+/CD4+ and CD3+/CD8+ cells, two indices of immune cells, were significantly higher in BD than in SC and BD^MSC^, and significantly higher in BD^MSC^ than in SC, whereas the circulating and spleen level of Treg+ cells (i.e., helps end an immune response), showed an opposite pattern of CD3+/CD4+ cells among the three groups.

### Protein expression of inflammatory and anti-inflammatory biomarkers in heart, liver and kidney by 6 h after the BD procedure

**Figure 2:**
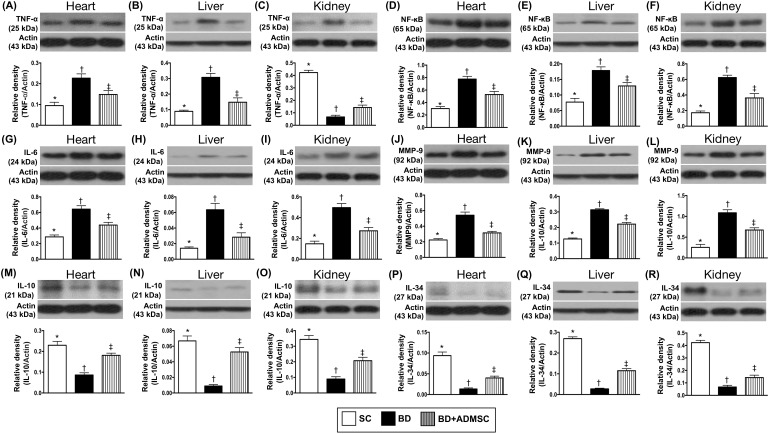
Protein expressions of inflammatory and anti-inflammatory biomarkers of heart, liver and kidney by 6 h after BD procedure **(A)** Protein expression of tumor necrosis factor (TNF)-α in heart, * vs. other groups with different symbols (†, ‡), p<0.001. **(B)** Protein expression of TNF-α in liver, * vs. other groups with different symbols (†, ‡), p<0.001. **(C)** Protein expression of TNF-α in kidney, * vs. other groups with different symbols (†, ‡), p<0.0001. **(D)** Protein expression of nuclear factor (NF)- κB in heart, * vs. other groups with different symbols (†, ‡), p<0.001. **(E)** Protein expression of NF- κB in liver, * vs. other groups with different symbols (†, ‡), p<0.001. **(F)** Protein expression of NF- κB in kidney, * vs. other groups with different symbols (†, ‡), p<0.001. **(G)** Protein expression of interleukin [6]-6 in heart, * vs. other groups with different symbols (†, ‡), p<0.001. **(H)** Protein expression of IL-6 in liver, * vs. other groups with different symbols (†, ‡), p<0.001. **(I)** Protein expression of IL-6 in kidney, * vs. other groups with different symbols (†, ‡), p<0.001. **(J)** Protein expression of matrix metalloproteinase (MMP)-9 in heart, * vs. other groups with different symbols (†, ‡), p<0.001. **(K)** Protein expression of MMP-9 in liver, * vs. other groups with different symbols (†, ‡), p<0.001. **(L)** Protein expression of MMP-9 in kidney, * vs. other groups with different symbols (†, ‡), p<0.001. **(M)** Protein expression of IL-10 in heart, * vs. other groups with different symbols (†, ‡), p<0.001. **(N)** Protein expression of IL-10 in liver, * vs. other groups with different symbols (†, ‡), p<0.0001. **(O)** Protein expression of IL-10 in kidney, * vs. other groups with different symbols (†, ‡), p<0.001. **(P)** Protein expression of IL-34 in heart, * vs. other groups with different symbols (†, ‡), p<0.001. **(Q)** Protein expression of IL-34 in liver, * vs. other groups with different symbols0 (†, ‡), p<0.0001. **(R)** Protein expression of IL-34 in kidney, * vs. other groups with different symbols (†, ‡), p<0.0001. All statistical analyses were performed by one-way ANOVA, followed by Bonferroni multiple comparison post hoc test (n=8). Symbols (*, †, ‡) indicate significance (at 0.05 level). BD = brain death; group A1 = sham control; group A2 = brain death; group A3 = brain death + adipose-derived mesenchymal stem cell (ADMSC).

Additionally, Western blot analysis was also performed for determining the proinflammatory biomarkers in heart, liver and kidney. The results exhibited that the protein expressions of TNF-α, NF-κB, IL-6 and matrix metalloproteinase (MMP)-9, four indicators of inflammation in the liver, kidney and heart were significantly higher in BD than in SC and BD^MSC^, and significantly higher in BD^MSC^ than in SC (Figure 2). On the other hand, the protein expression of IL-10 and IL-34, two indicators of anti-inflammation, showed an opposite pattern of inflammation among the three groups (Figure 2).

### Protein and cellular expression of inflammatory biomarkers in the brain at 6 h after BD procedure

**Figure 3:**
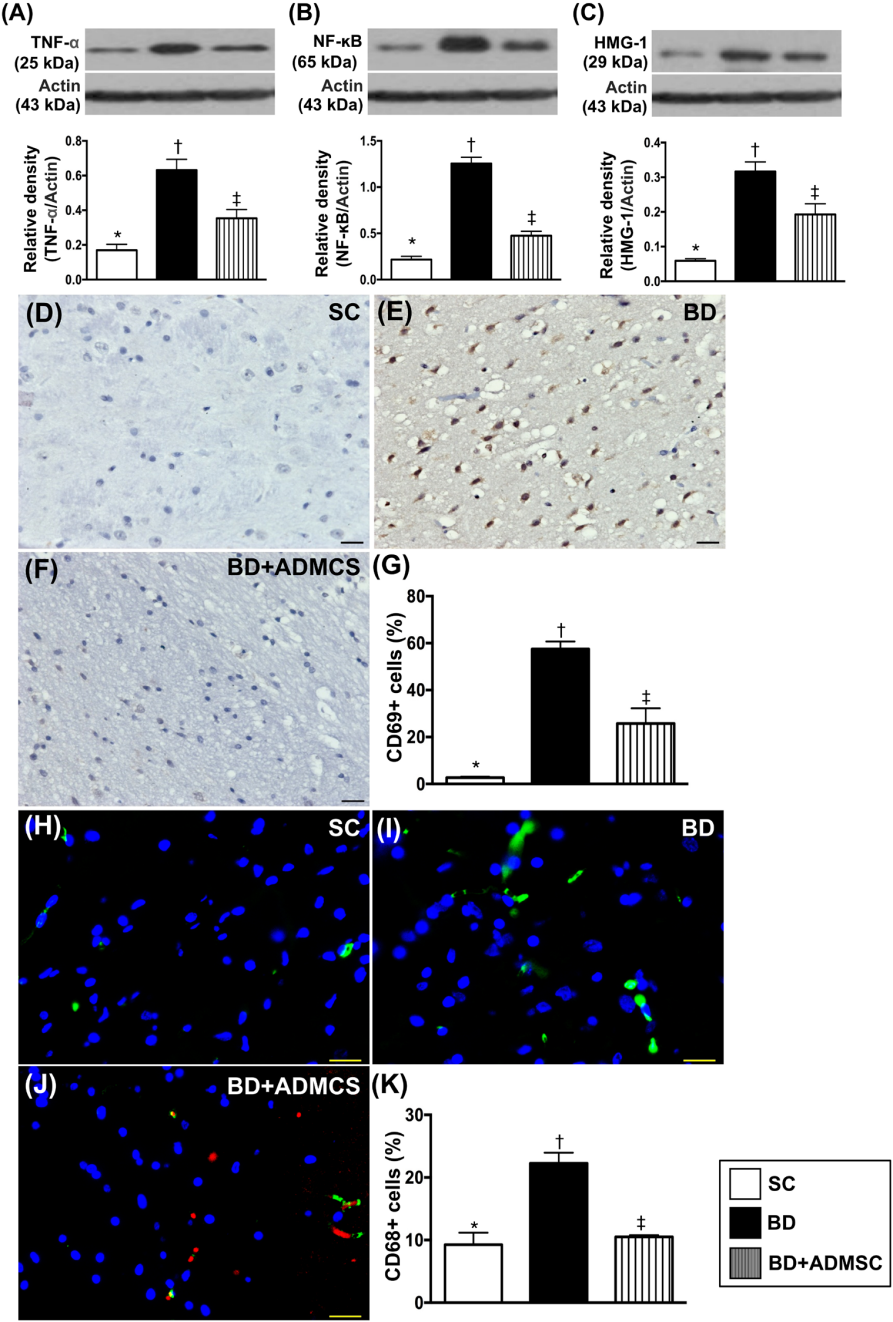
The protein and cellular expressions of inflammatory biomarkers in brain at 6 h after BD procedure **(A)** Protein expression of tumor necrosis factor (TNF)-α, * vs. other bars with different symbols, p<0.0001. **(B)** Protein expression of nuclear factor (NF)-κB, * vs. other groups with different symbols (†, ‡), p<0.0001. **(C)** Protein expression of high-mobility group protein-1 (HMG-1), * vs. other groups with different symbols (†, ‡), p<0.0001. **(D** to **F)** Illustrating microscopic finding (200x) of immunohistochemical staining for identification of CD69+ cells (gray color). The scale bars in right lower corner represent 50 μm. **(G)** Analytical result of number of positively-stained CD69 cells, * vs. other groups with different symbols (†, ‡), p<0.001. **(H** to **J)** Illustrating microscopic finding (400x) of immunofluorescent staining for identification of F4/80+ cells (green color). Red color in [1] indicated Dil-dye positively-stained ADMSCs in brain tissue. The scale bars in right lower corner represent 20 μm. **(K)** Analytical result of number of positively-stained F4/80 cells, * vs. other groups with different symbols (†, ‡), p<0.001. All statistical analyses were performed by one-way ANOVA, followed by Bonferroni multiple comparison post hoc test (n=8). Symbols (*, †, ‡) indicate significance (at 0.05 level). BD = brain death; group A1 = sham control; group A2 = brain death; group A3 = brain death + adipose-derived mesenchymal stem cell (ADMSC).

To measure the proinflammatory biomarkers in the brain tissue, Western blot analysis was used. The results showed that the protein expression of TNF-α and nuclear factor (NF)-κB, two indicators of inflammation and high mobility group protein-1 (HMG-1), an indicator of cytokine mediator of inflammation, were higher in BD than in SC and BD^MSC^, and significantly higher in BD^MSC^ than in SC (Figure 3). Additionally, cellular expressions of CD69+ and F4/80+ cells (i.e., a macrophage surface marker), two indices of inflammation, exhibited an identical pattern of protein expression of inflammation among the three groups (Figure 3).

### Cellular expression of inflammatory biomarkers in the heart, liver and kidney at 6 h after the BD procedure

**Figure 4:**
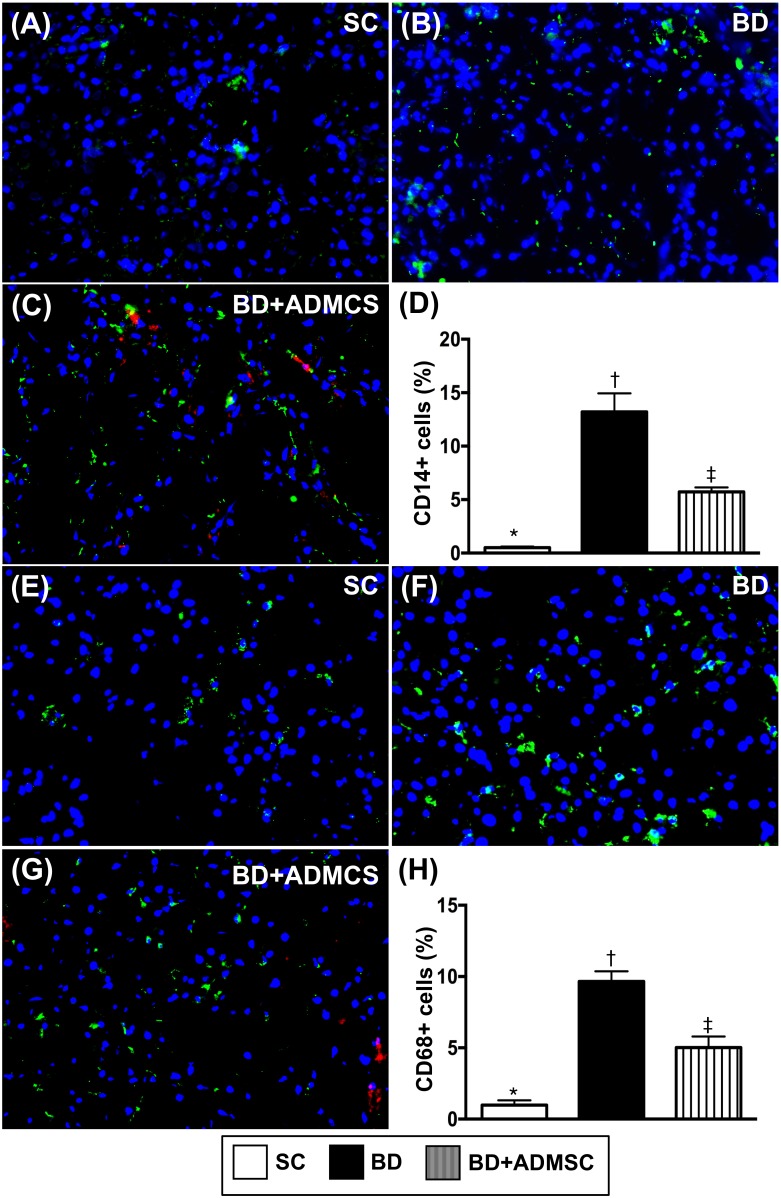
Cellular expressions of inflammatory biomarkers in heart at 6 h after BD procedure **(A-C)** Illustrating immunofluorescent (IF) microscopic finding (400x) for identifying CD14+ cells (green color). Red color in [4] indicated some Dil-dye positively-stained ADMSCs in heart tissue. **(D)** Analytical result of number of positively-stained CD cells, * vs. other groups with different symbols (†, ‡), p<0.0001. **(E** to **G)** IF microscopic finding (400x) for identifying CD68+ cells (green color). Red color in (G) indicated some Dil-dye positively-stained ADMSCs in heart tissue. **(H)** Analytical result of number of positively-stained CD68+ cells, * vs. other groups with different symbols (†, ‡), p<0.0001. The scale bars in right lower corner represent 20 μm. All statistical analyses were performed by one-way ANOVA, followed by Bonferroni multiple comparison post hoc test (n=8). Symbols (*, †, ‡) indicate significance (at 0.05 level). BD = brain death; group A1 = sham control; group A2 = brain death; group A3 = brain death + adipose-derived mesenchymal stem cell (ADMSC).

**Figure 5:**
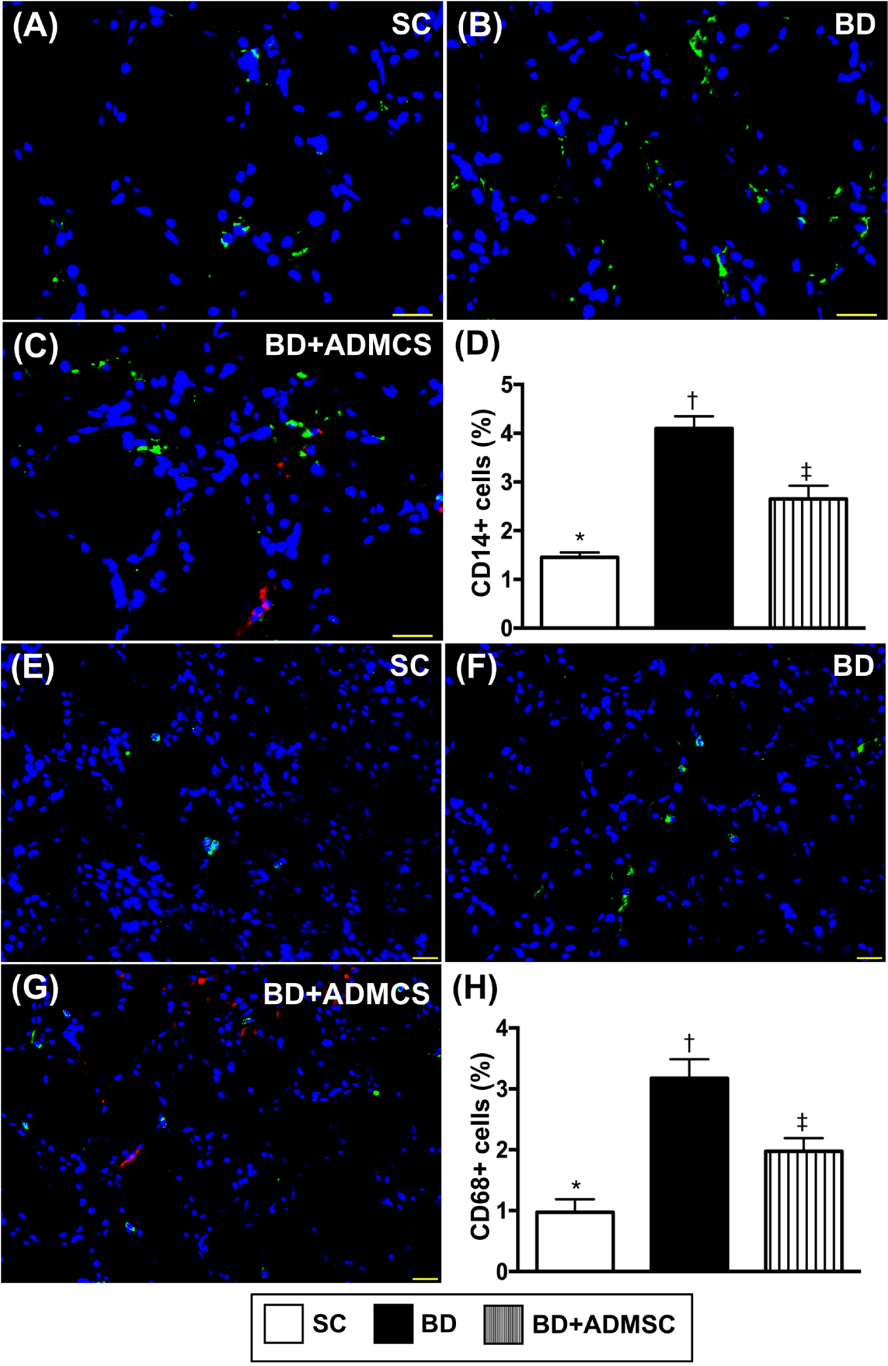
Cellular expressions of inflammatory biomarkers in liver at 6 h after BD procedure **(A** to **C)** Illustrating immunofluorescent (IF) microscopic finding (400x) for identifying CD14+ cells (green color). Red color in [4] indicated some Dil-dye positively-stained ADMSCs in heart tissue. **(D)** Analytical result of number of positively-stained CD14 cells, * vs. other groups with different symbols (†, ‡), p<0.0001. **(E** to **G)** IF microscopic finding (400x) for identifying CD68+ cells (green color). Red color in [4] indicated some Dil-dye positively-stained ADMSCs in heart tissue. **(H)** Analytical result of number of positively-stained CD68+ cells, * vs. other groups with different symbols (†, ‡), p<0.0001. The scale bars in right lower corner represent 20 μm. All statistical analyses were performed by one-way ANOVA, followed by Bonferroni multiple comparison post hoc test (n=8). Symbols (*, †, ‡) indicate significance (at 0.05 level). BD = brain death; group A1 = sham control; group A2 = brain death; group A3 = brain death + adipose-derived mesenchymal stem cell (ADMSC).

**Figure 6:**
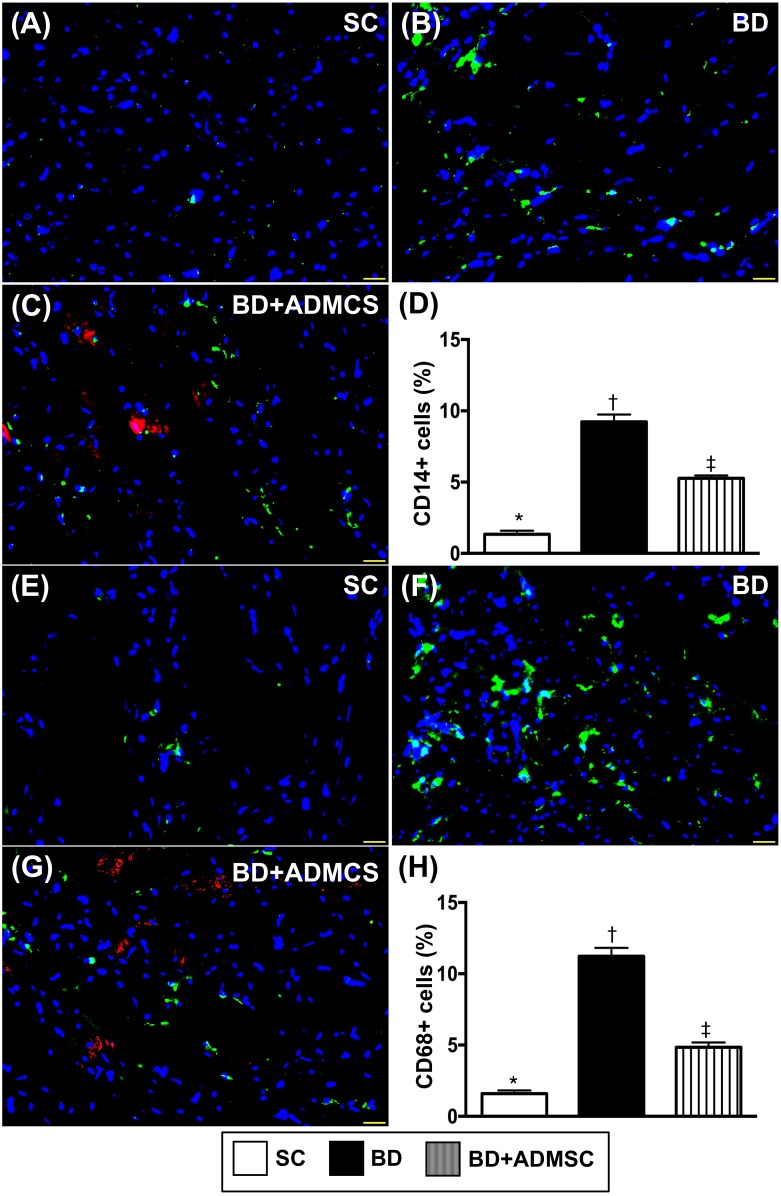
Cellular expressions of inflammatory biomarkers in kidney at 6 h after BD procedure **(A** to **C)** Illustrating immunofluorescent (IF) microscopic finding (400x) for identifying CD14+ cells (green color). Red color in [4] indicated some Dil-dye positively-stained ADMSCs in heart tissue. **(D)** Analytical result of number of positively-stained CD cells, * vs. other bars with different symbols, p<0.0001. **(E** to **G)** IF microscopic finding (400x) for identifying 68+ cells (green color). Red color in [4] indicated some Dil-dye positively-stained ADMSCs in heart tissue. **H)** Analytical result of number of positively-stained CD68+ cells, * vs. other groups with different symbols (†, ‡), p<0.0001. The scale bars in right lower corner represent 20 μm. All statistical analyses were performed by one-way ANOVA, followed by Bonferroni multiple comparison post hoc test (n=8). Symbols (*, †, ‡) indicate significance (at 0.05 level). BD = brain death; group A1 = sham control; group A2 = brain death; group A3 = brain death + adipose-derived mesenchymal stem cell (ADMSC).

Microscopy was utilized for identification of inflammatory cell expressions in heart, liver and kidney. The cellular expressions of CD14+ and CD68+ cells, two indices of inflammation in the liver (Figure 4), kidney (Figure 5) and heart (Figure 6), were significantly higher in BD than in SC and BD^MSC^, and significantly higher in BD^MSC^ than in SC.

### Anatomical-pathological findings and echocardiographic findings prior to and at day 5 after BD

**Figure 7:**
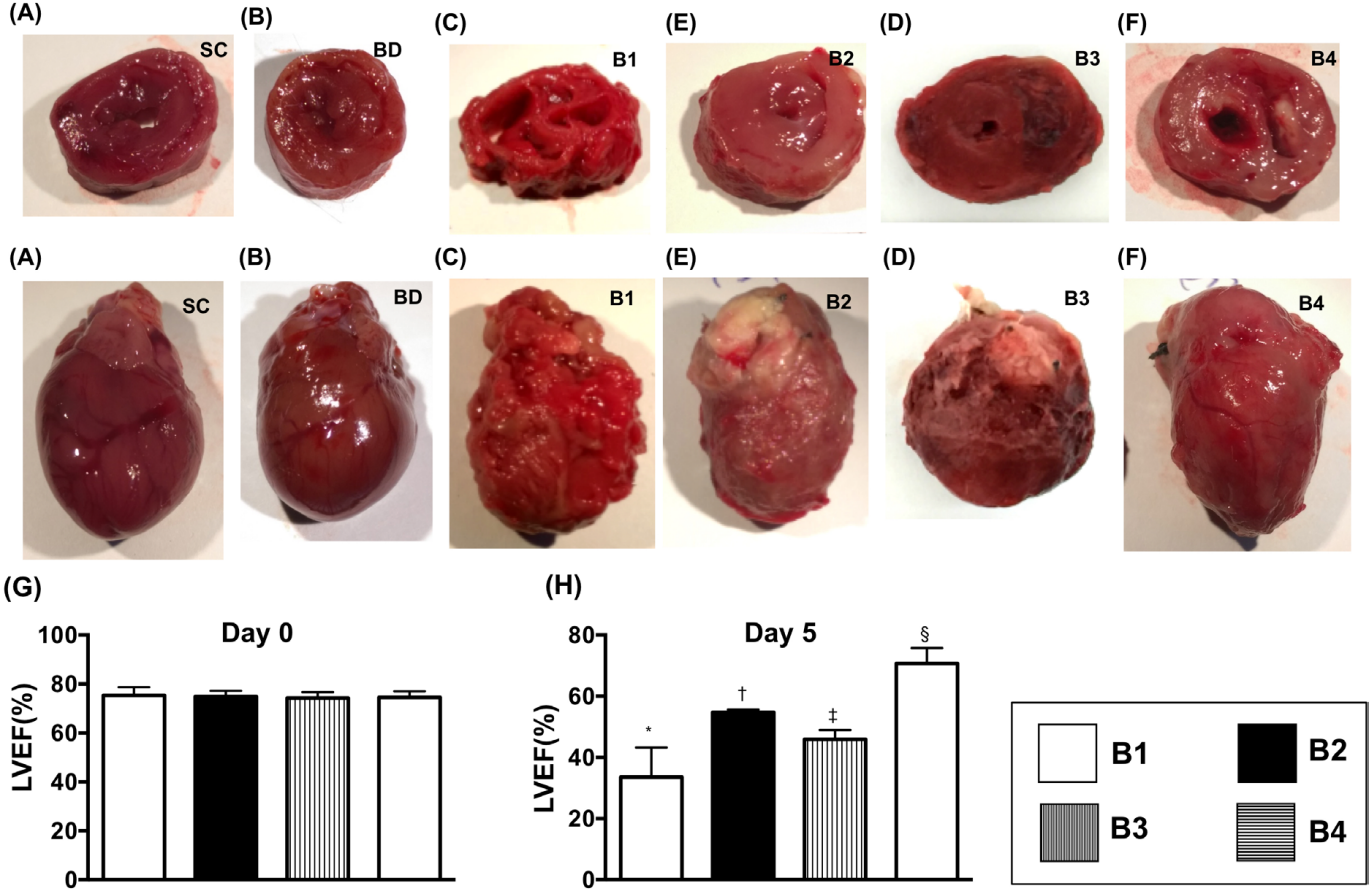
Anatomical-pathological findings and echocardiographic findings by day 5 after BD **(A** to **F)** Illustration of anatomical-pathological findings of the hearts (i.e., whole heart and cross section) in groups: sham control **(A)**, BD animal [1], donor heart of BD animal (i.e., Fischer344=F344) transplanted into recipient (i.e., Lewis) without treatment [4], F344 heart transplanted to Lewis + ADMSC administration at days 1 and 3 post-transplanted procedure **(D)**, F344 heart transplanted to Lewis + ADMSC administration at 3 h after BD procedure **(E)**, and F344 heart transplanted to Lewis + ADMSC administration at 3 h after BD and by days 1 and 3 after heart transplanted procedure [3]. The pathological findings showed that as compared with the other groups, the transplanted heart [4] was severely destroyed at day 5 after transplantation procedure, suggesting an occurrence of acute transplanted heart rejection. **(G** and **H)** Transthoracic echocardiographic findings for identification of left ventricular ejection fraction (LVEF). By day 0 (G): p>0.5. By day 5 [1], * vs. other groups with different symbols (†, ‡, §), p<0.0001. All statistical analyses were performed by one-way ANOVA, followed by Bonferroni multiple comparison post hoc test (n=8). Symbols (*, †, ‡, §) indicate significance (at 0.05 level). B1 = F344 heart transplanted into Lewis by 6 h after BD; B2 = BD induction for 6 h followed by heart transplantation and ADMSC (1.2x10^6^ cells) transfused into recipient at days 1 and 3, respectively after transplantation; B3 **=** BD + ADMSC (1.2x10^6^ cells) at 3 h and heart transplantation at 6 h after BD; B4 = BD + ADMSC (1.2x10^6^ cells) at 3h and heart transplantation at 6 h after BD, followed by ADMSC (1.2x10^6^ cells) transfusion to recipient by days 1 and 3, respectively after heart transplantation. BD = brain death; ADMSC = adipose-derived mesenchymal stem cell.

Grossly anatomical structure of each heart was assessed in details. As expected, pathological findings showed that as compared to normal heart, the transplanted heart was severely destroyed at day 5 after the transplantation procedure, suggesting an occurrence of acute transplanted heart rejection (Figure 7). However, the transplanted heart was notably protected by ADMSC therapy at a time period of 3 h after the BD procedure (i.e., allogenic ADMSC administration to donor prior to transplantation) and furthermore protected by the ADMSC treatment just after the transplant procedure (i.e., allogenic ADMSC administration to the recipients, Lewis rats) as well as AMDS treatment at the two time points (i.e., prior to and after F344 heart transplantation to Lewis), implying that allogenic ADMSC therapy protected the transplanted heart from graft versus host disease (Figure 7).

For assessment of the functional changes of the heart, transthoracic echocardiographic examination was performed for each animal. As expected, prior to BD, the transthoracic echocardiographic examination showed that the LVEF did not differ among the four groups: BD-T (F344 heart transplanted into Lewis by 6h after BD), BD-T^MSC(D1/3)^ (BD induction for 6h then heart transplantation, and allogenic ADMSCs transfusion at days 1 and 3 after heart transplantation), BD-T^MSC(3h)^ (BD + ADMSC/1.2 × 10^6^ cells at 3h and heart transplantation at 6h after BD) and BD-T^MSC(3h, D1/3)^ [BD + ADMSC/1.2 × 10^6^ cells at 3h and heart transplantation at 6h after BD, then ADMSC therapy by days 1/3]. However, by day 5 prior to the animals being euthanized, this parameter was highest in BD-T^MSC(3h, D1/3)^, lowest in BD-T, and significantly higher in BD-T^MSC(D1/3)^ than in BD-T^MSC(3h)^, suggesting that allogenic ADMSC treatment protected the heart function against acute immune rejection-induced damage (Figure 7).

### Flow cytometric analysis of inflammatory cells in circulation and in the spleen and protein expression of apoptotic, fibrotic and anti-fibrotic biomarkers in transplanted LV myocardium by day 5 after heart transplantation

**Figure 8:**
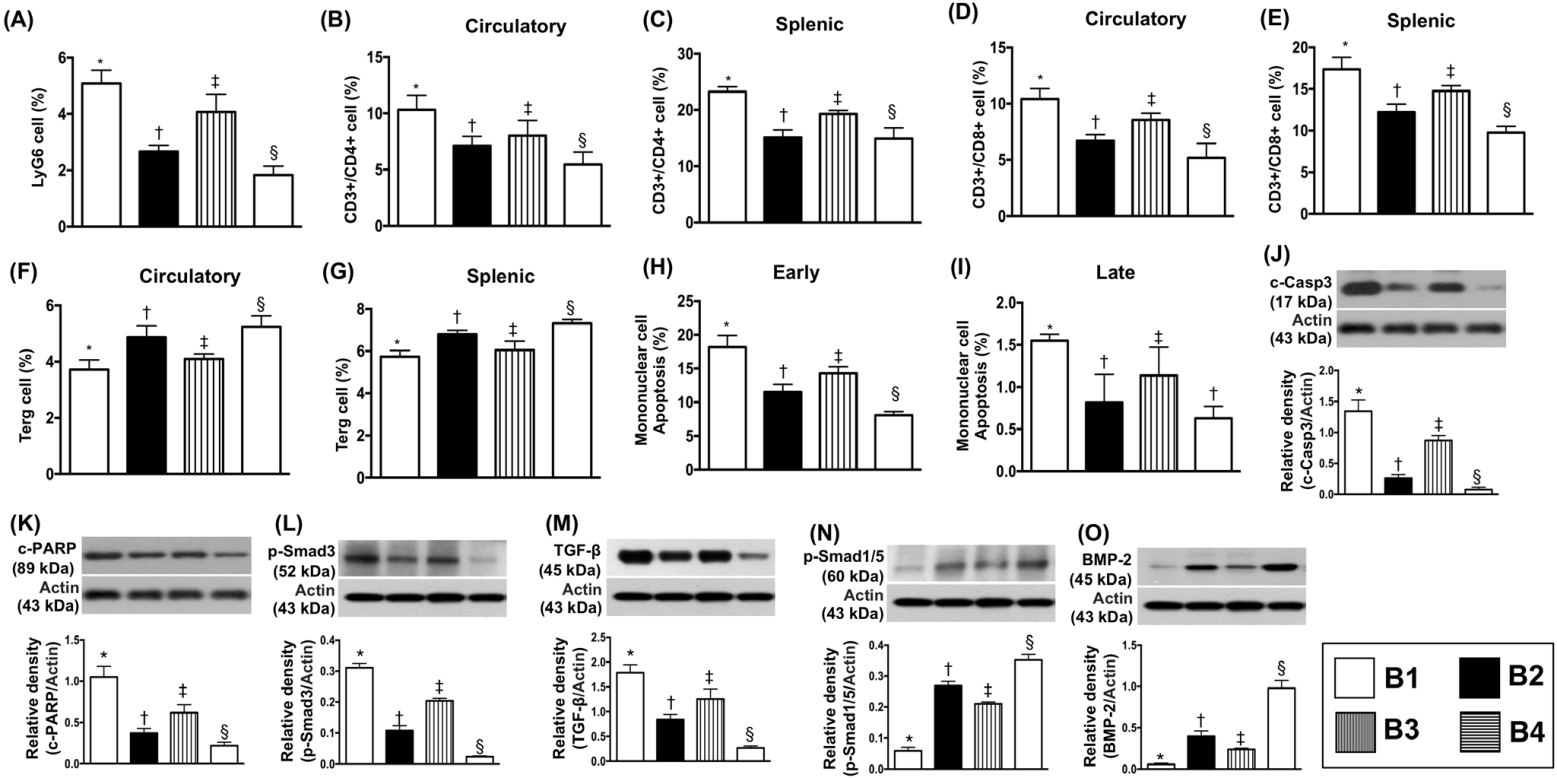
Flow cytometric analysis of inflammatory cells in circulation and spleen and the protein expressions of apoptotic, fibrotic and anti-fibrotic biomarkers by day 5 after heart transplantation **(A)** Circulating level of Ly6G, * vs. other groups with different symbols (†, ‡, §), p<0.001. **(B)** Circulating levels of CD3+/CD4+ cells, * vs. other groups with different symbols (†, ‡, §), p<0.001. **(C)** Splenic levels of CD3+/CD4+ cells, * vs. other groups with different symbols (†, ‡, §), p<0.001. **(D)** Circulating levels of CD3+/CD8+ cells, * vs. other groups with different symbols (†, ‡, §), p<0.001. **(E)** Splenic levels of CD3+/CD8+ cells, * vs. other groups with different symbols (†, ‡, §), p<0.001. **(F)** Circulating levels of Treg cells (CD4+CD25+Foxp3+ cells), * vs. other bars with different symbols (†, ‡, §), p<0.001. **(G)** Splenic levels of Treg cells, * vs. other groups with different symbols (†, ‡, §), p<0.001, p<0.001. **(H)** Early apoptosis, * vs. other groups with different symbols (†, ‡, §), p<0.001. **(I)** Late apoptosis, * vs. other groups with different symbols (†, ‡), p<0.001. **(J)** Protein expression of cleaved caspase 3 (c-Casp 3), * vs. other groups with different symbols (†, ‡, §), p<0.0001. **(K)** Protein expression of cleaved poly (ADP-ribose) polymerase (c-PARP), * vs. other groups with different symbols (†, ‡, §), p<0.0001. **(L)** Protein expression of phosphorylated (p)-Smad3, * vs. other groups with different symbols (†, ‡, §), p<0.0001. **(M)** Protein expression of transforming growth factor (TGF)-ß, * vs. other groups with different symbols (†, ‡, §), p<0.0001. **(N)** Protein expression of p-Smad1/5, * vs. other groups with different symbols (†, ‡, §), p<0.0001. **(O)** Protein expression of bone morphogenetic protein (BMP)-2, * vs. other groups with different symbols (†, ‡, §), p<0.0001. All statistical analyses were performed by one-way ANOVA, followed by Bonferroni multiple comparison post hoc test (n=8). Symbols (*, †, ‡, §) indicate significance (at 0.05 level). B1 = F344 heart transplanted into Lewis by 6 h after BD; B2 = BD induction for 6 h followed by heart transplantation and ADMSC (1.2x10^6^ cells) transfused into recipient at days 1 and 3, respectively after transplantation; B3 = BD + ADMSC (1.2x10^6^ cells) at 3 h and heart transplantation at 6 h after BD; B4 = BD + ADMSC (1.2x10^6^ cells) at 3h and heart transplantation at 6 h after BD, followed by ADMSC (1.2x10^6^ cells) transfusion to recipient by days 1 and 3, respectively after heart transplantation. BD = brain death; ADMSC = adipose-derived mesenchymal stem cell.

The circulating level of Ly6G+ cells, an indicator of inflammation, was highest in BD-T and lowest in BD-T^MSC(3h, D1/3)^, and significantly higher in BD-T^MSC(3h)^ than in BD-T^MSC(D1/3)^. Additionally, circulating and splenic levels of CD3+/CD4+ and CD3+/CD8+ cells (i.e., helps end an immune response) expressed an identical pattern, whereas Treg+ cells (i.e., an indicator of T-helper cells) exhibited an opposite pattern of Ly6G+ cells among the four groups (Figure 8).

The flow cytometric analysis showed that the early apoptotic cells were highest in BD-T and lowest in BD-T^MSC(3h, D1/3)^, significantly higher in BD-T^MSC(3h)^ than in BD-T^MSC(D1/3)^. Additionally, the late apoptotic cells displayed a similar pattern of early apoptosis except for no difference between BD-T^MSC(D1/3)^ and BD-T^MSC(3h)^ (Figure 8).

The protein expressions of cleaved caspase 3 and cleaved poly(ADP-ribose) polymerase (PARP), two indicators of apoptosis, and protein expressions of Smad3 and transforming growth factor (TGF)-β, two indicators of fibrosis, displayed an identical pattern, whereas the protein expressions of Smad1/5 and bone morphogenetic protein-2 (BMP-2), two indicators of anti-fibrosis, revealed an opposite pattern of Ly6G+ cells among the four groups (Figure 8).

### Protein expressions of inflammatory biomarkers in the transplanted heart by day 5 after BD procedure

**Figure 9:**
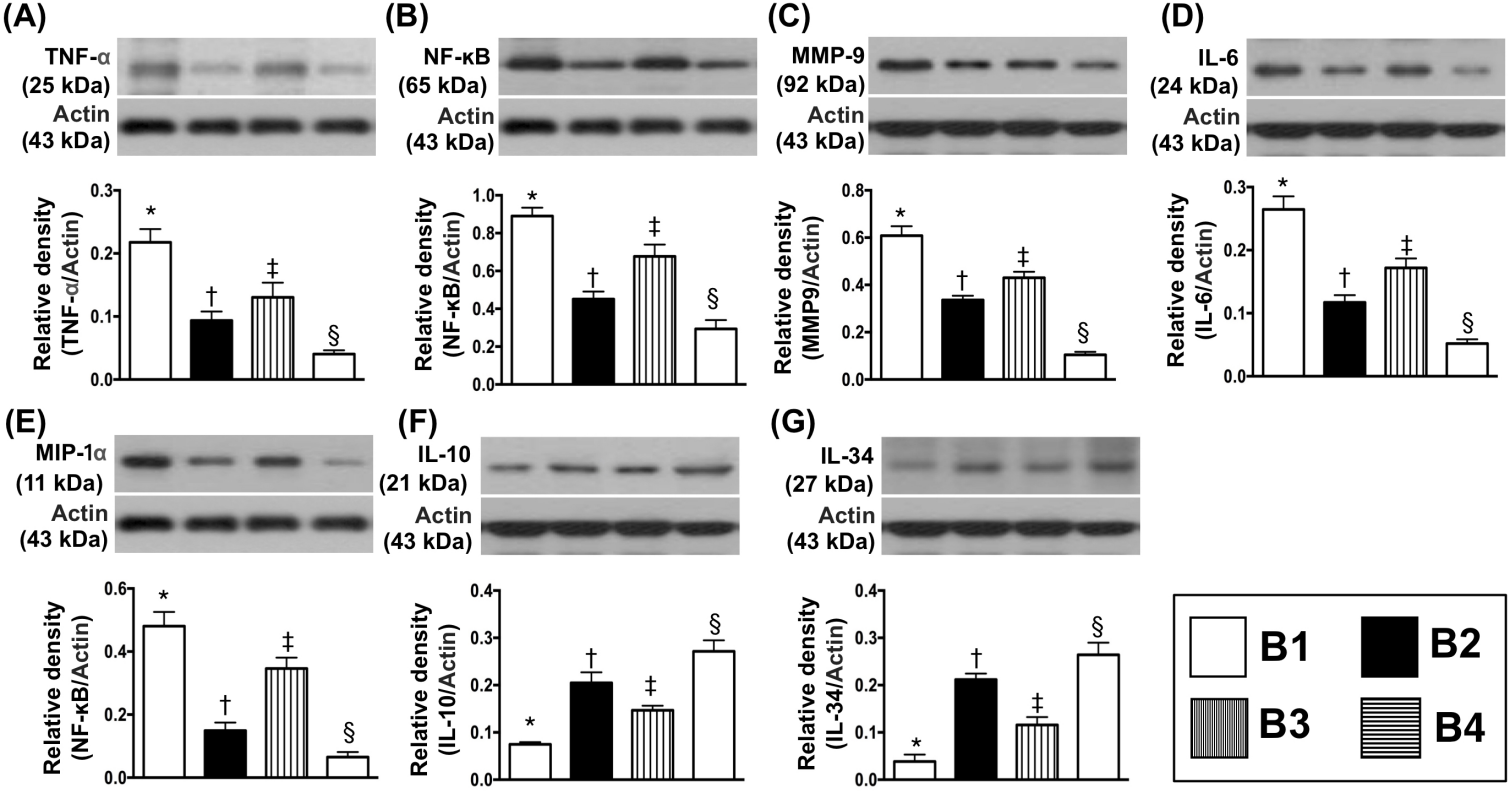
Protein expressions of inflammatory biomarkers in transplanted heart by day 5 after heart transplantation **(A)** Protein expression of tumor necrosis factor (TNF)-α, * vs. other groups with different symbols (†, ‡, §), p<0.001. **(B)** Protein expression of nuclear factor (NF)- κB, * vs. other groups with different symbols (†, ‡, §), p<0.0001. **(C)** Protein expression of matrix metalloproteinase (MMP)-9, * vs. other groups with different symbols (†, ‡, §), p<0.0001. **(D)** Protein expression of interleukin [6]-6, * vs. other groups with different symbols (†, ‡, §), p<0.0001. **(E)** Protein expression of macrophage inflammatory protein (MIP)-1α, * vs. other groups with different symbols (†, ‡, §), p<0.0001. **(F)** Protein expression of IL-10, * vs. other groups with different symbols (†, ‡, §), p<0.0001. **(G)** Protein expression of IL-34, * vs. other groups with different symbols (†, ‡, §), p<0.0001. All statistical analyses were performed by one-way ANOVA, followed by Bonferroni multiple comparison post hoc test (n=8). Symbols (*, †, ‡, §) indicate significance (at 0.05 level). B1 = F344 heart transplanted into Lewis by 6 h after BD; B2 = BD induction for 6 h followed by heart transplantation and ADMSC (1.2x10^6^ cells) transfused into recipient at days 1 and 3, respectively after transplantation; B3 **=** BD + ADMSC (1.2x10^6^ cells) at 3 h and heart transplantation at 6 h after BD; B4 = BD + ADMSC (1.2x10^6^ cells) at 3h and heart transplantation at 6 h after BD, followed by ADMSC (1.2x10^6^ cells) transfusion to recipient by days 1 and 3, respectively after heart transplantation. BD = brain death; ADMSC = adipose-derived mesenchymal stem cell.

The protein expressions of TNF-α, NF-κB, MMP-9, IL-6 and macrophage inflammatory protein (MIP)-1α, five indices of inflammation were highest in BD-T and lowest in BD-T^MSC(3h, D1/3)^, and significantly higher in BD-T^MSC(3h)^ than in BD-T^MSC(D1/3)^. On the other hand, the protein expressions of IL-10 and IL-34 showed an opposite pattern of inflammatory biomarkers among the four groups (Figure 9).

### Histopathological findings and DNA-damage markers in transplanted heart by day 5 after BD procedure

**Figure 10:**
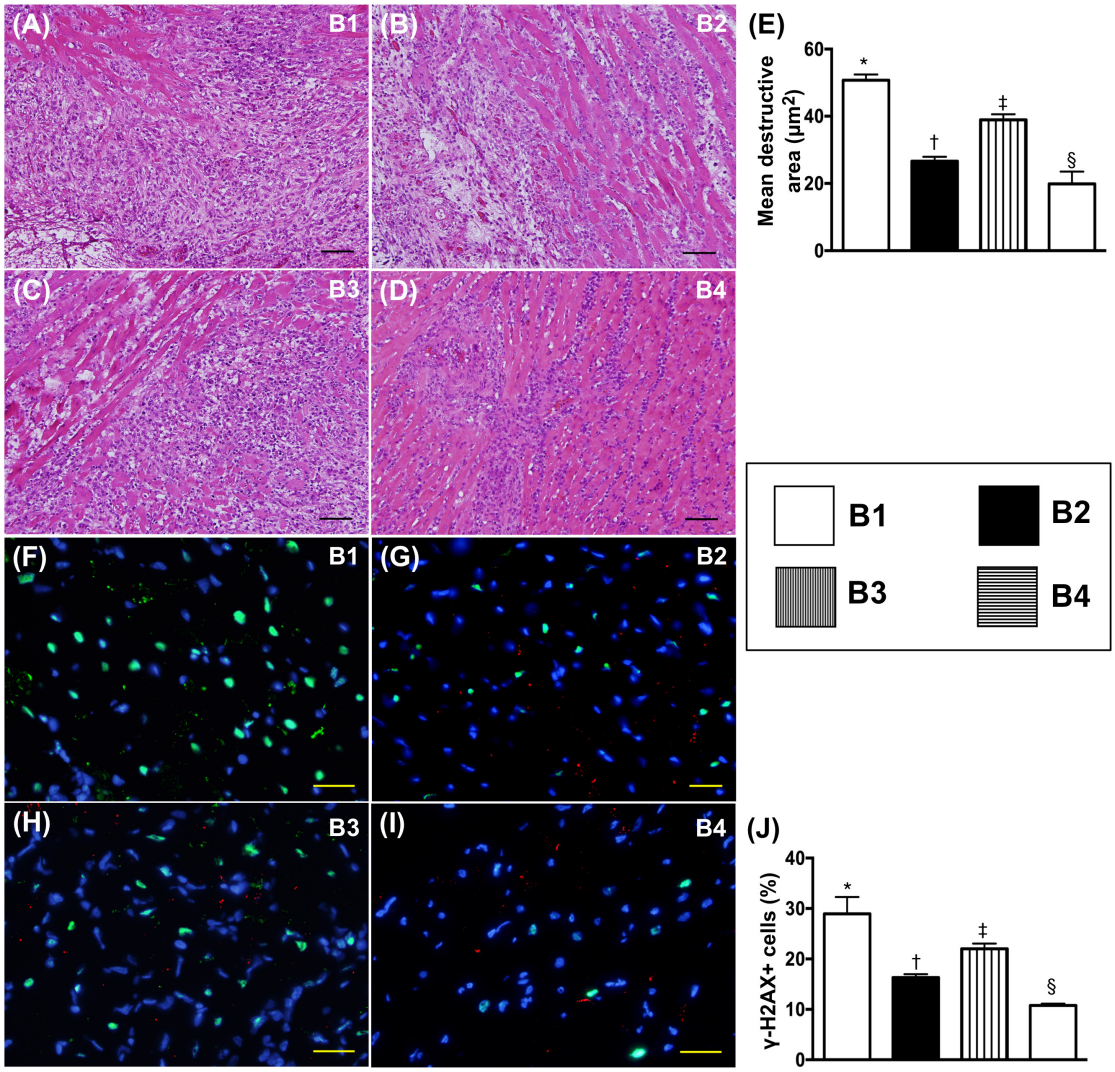
Histopathological finding and DNA-damaged marker in transplanted heart by day 5 after heart transplantation **(A** to **D)** Illustrating the light microscopic finding (100x) of H & E stain for identifying the destructive area (blue-colored fibrotic area). The scale bars in right lower corner represent 100 μm. **(E)** Analytic result of the destructive area, * vs. other groups with different symbols (†, ‡, §), p<0.0001. **(F** to **I)** Illustrating the microscopic finding (400x) of immunofluorescent stain for identification of γ-H2AX+ cells (green color). The scale bars in right lower corner represent 20 μm. **(J)** Analytic results of number of positively-stained γ-H2AX cells, * vs. other groups with different symbols (†, ‡, §), p<0.0001. All statistical analyses were performed by one-way ANOVA, followed by Bonferroni multiple comparison post hoc test (n=8). Symbols (*, †, ‡, §) indicate significance (at 0.05 level). B1 = F344 heart transplanted into Lewis by 6 h after BD; B2 = BD induction for 6 h followed by heart transplantation and ADMSC (1.2x10^6^ cells) transfused into recipient at days 1 and 3, respectively after transplantation; B3 **=** BD + ADMSC (1.2x10^6^ cells) at 3 h and heart transplantation at 6 h after BD; B4 = BD + ADMSC (1.2x10^6^ cells) at 3h and heart transplantation at 6 h after BD, followed by ADMSC (1.2x10^6^ cells) transfusion to recipient by days 1 and 3, respectively after heart transplantation. BD = brain death; ADMSC = adipose-derived mesenchymal stem cell.

The light microscopic finding of hematoxylin and eosin (H&E) stain showed that the integrity of myocardial architecture per high-power field was lowest in BD-T and highest in BD-T^MSC(3h, D1/3)^, and significantly lower in BD-T^MSC(3h)^ than in BD-T^MSC(D1/3)^, shedding light on the impact of ADMSC treatment on suppressing the frequency of post-heart transplanted rejection. On the other hand, the cellular expression of γ-H2AX+ cells, an indicator of DNA-damage, displayed an opposite pattern of integrity of myocardial architecture among the four groups (Figure 10).

### Cellular expressions of inflammatory biomarkers in transplanted heart by day 5 after BD procedure

**Figure 11:**
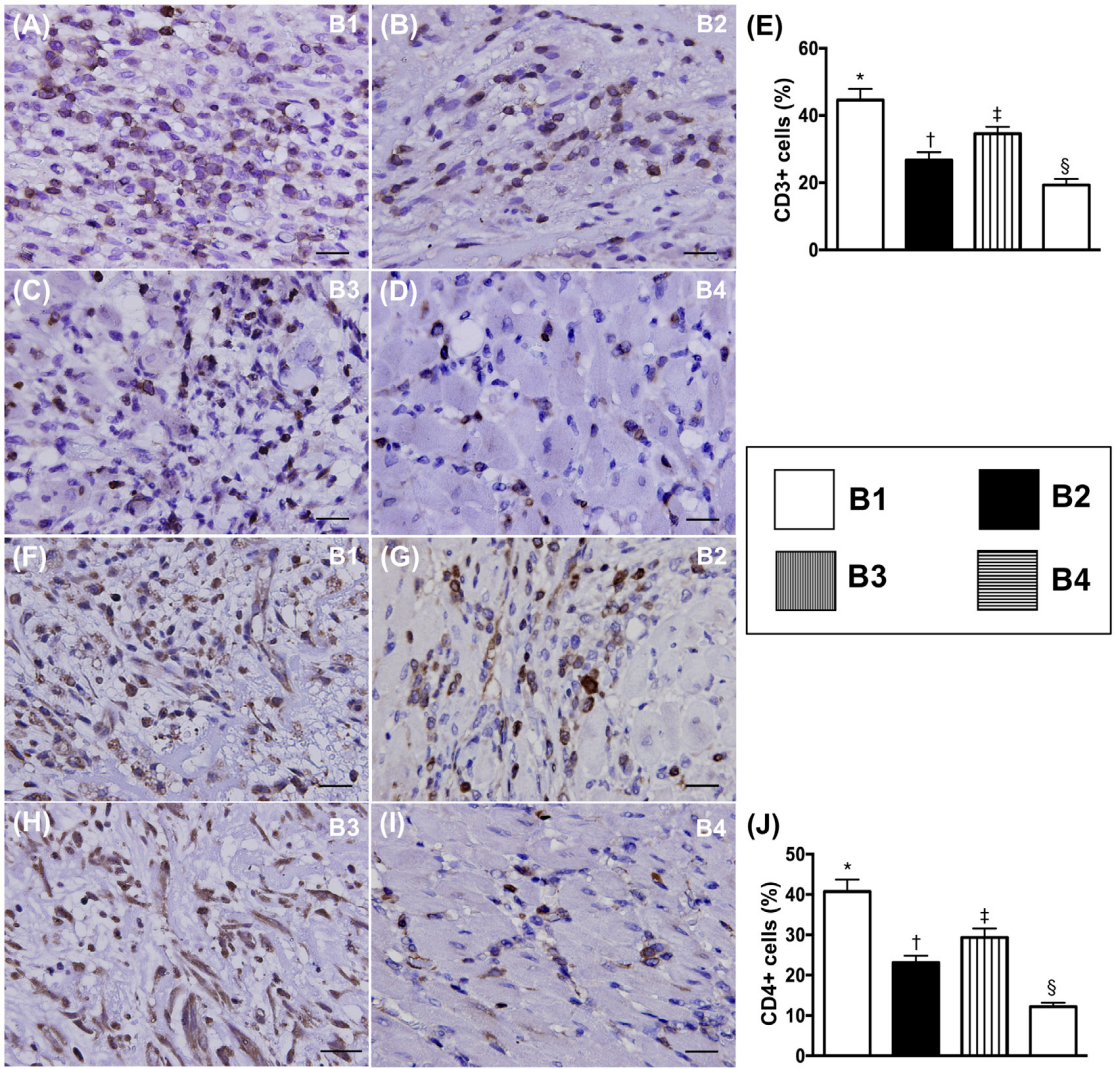
Immune cell expression in transplanted heart by day 5 after heart transplantation **(A** to **D)** Illustrating microscopic finding (400x) of immunohistochemical (IHC) staining for identification of CD3+ cells (gray color). **(E)** Analytical results of number of positively-stained CD3 cells, * vs. other groups with different symbols (†, ‡, §), p<0.001. **(F** to **I)** Illustrating microscopic finding of IHC staining for identification of CD4+ cells (gray color). **(J)** Analytical results of number of positively-stained CD4 cells, * vs. other groups with different symbols (†, ‡, §), p<0.001. The scale bars in right lower corner represent 20 μm. All statistical analyses were performed by one-way ANOVA, followed by Bonferroni multiple comparison post hoc test (n=8). Symbols (*, †, ‡, §) indicate significance (at 0.05 level). B1 = F344 heart transplanted into Lewis by 6 h after BD; B2 = BD induction for 6 h followed by heart transplantation and ADMSC (1.2x10^6^ cells) transfused into recipient at days 1 and 3, respectively after transplantation; B3 **=** BD + ADMSC (1.2x10^6^ cells) at 3 h and heart transplantation at 6 h after BD; B4 = BD + ADMSC (1.2x10^6^ cells) at 3h and heart transplantation at 6 h after BD, followed by ADMSC (1.2x10^6^ cells) transfusion to recipient by days 1 and 3, respectively after heart transplantation. BD = brain death; ADMSC = adipose-derived mesenchymal stem cell.

The immunofluorescence (IF) microscopic findings demonstrated that the number of CD14+ and F4/80+ cells, two indicators of inflammation, were higher in BD-T and lowest in BD-T^MSC(3h, D1/3)^, and significantly higher in BD-T^MSC(3h)^ than in BD-T^MSC(D1/3)^ (Figure 11).

### Expression of immune cells in transplanted heart by day 5 after BD procedure

**Figure 12:**
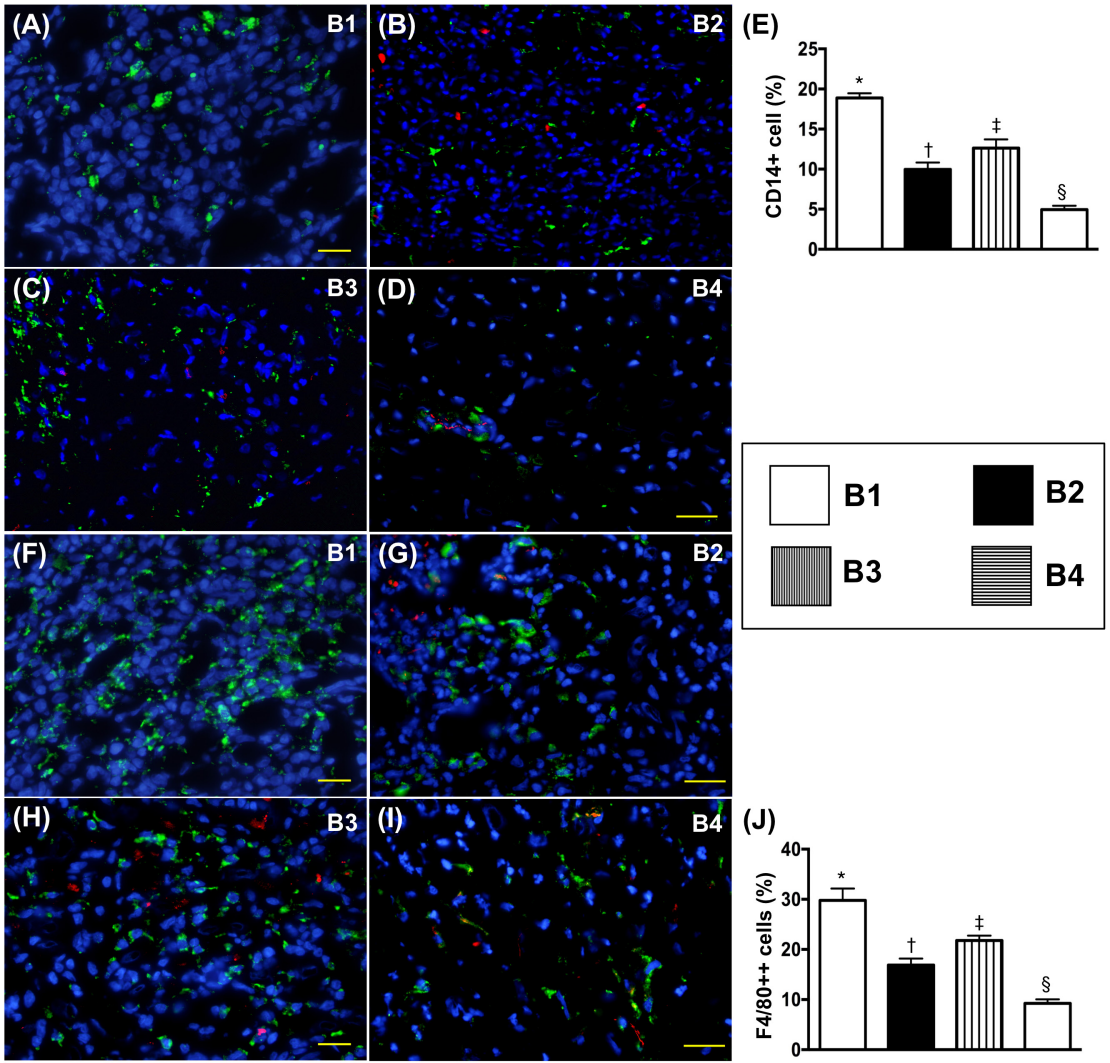
Cellular expressions of inflammatory biomarkers in transplanted heart by day 5 after heart transplantation **(A** to **D)** Illustrating microscopic finding (400x) of immunofluorescent (IF) staining for identification of CD14+ cells (green color). Red color in (B, C, D) indicated some Dil-dye positively-stained ADMSCs in heart tissue. **(E)** Analytical results of number of positively-stained CD14 cells, * vs. other groups with different symbols (†, ‡, §), p<0.0001. **(F** to **I)** Illustrating microscopic finding of IF staining for identification of F4/80+ cells (green color). Red color in (G, H, I) indicated some Dil-dye positively-stained ADMSCs in heart tissue. **(J)** Analytical results of number of positively-stained F4/80 cells, * vs. other groups with different symbols (†, ‡, §), p<0.0001. The scale bars in right lower corner represent 20 μm. All statistical analyses were performed by one-way ANOVA, followed by Bonferroni multiple comparison post hoc test (n=8). Symbols (*, †, ‡, §) indicate significance (at 0.05 level). B1 = F344 heart transplanted into Lewis by 6 h after BD; B2 = BD induction for 6 h followed by heart transplantation and ADMSC (1.2x10^6^ cells) transfused into recipient at days 1 and 3, respectively after transplantation; B3 **=** BD + ADMSC (1.2x10^6^ cells) at 3 h and heart transplantation at 6 h after BD; B4 = BD + ADMSC (1.2x10^6^ cells) at 3h and heart transplantation at 6 h after BD, followed by ADMSC (1.2x10^6^ cells) transfusion to recipient by days 1 and 3, respectively after heart transplantation. BD = brain death; ADMSC = adipose-derived mesenchymal stem cell.

Immunohistochemical (IHC) microscopic findings showed that the cellular expressions of CD3+ and CD4+ cells, two indicators of immune cells, were highest in BD-T and lowest in BD-T^MSC(3h, D1/3)^, and significantly higher in BD-T^MSC(3h)^ than in BD-T^MSC(D1/3)^ (Figure 12).

## DISCUSSION

This study which investigated the impact of BD on remote organ damage and the therapeutic role of ADMSCs in protecting BD-induced remote organ damage and the transplanted heart from graft versus host disease yielded several striking results. Frist, BD was identified not only to elicit inflammatory/immune reactions but also to cause remote organ damage. Second, allogenic ADMSC therapy markedly suppressed BD-caused remote organ damage. Third, allogenic ADMSC therapy protected the transplanted heart from graft versus host disease.

Intriguingly, experimental studies have shown that a vigorous inflammatory reaction and hyper-reactive immune response frequently occur in major organs of BD animals [34–36]. Another essential finding in the present study was that the protein levels of inflammation in the brain and both protein and cellular levels of inflammation in three major organs (i.e., liver, kidney and heart) were substantially enhanced in the BD group as compared to the control group. However, these molecular-cellular perturbations were remarkably attenuated in BD animals after receiving ADMSC treatment. Our findings, therefore, in addition to reinforcing the findings from previous studies [34–36], highlight the potential role of MSC therapy in clinical settings of graft versus host disease.

It is well known that the legal donors for heart transplantation are always BD victims. The donor heart is a precious gift for the recipient. Interestingly, not only inflammatory and immune reactions but also heart failure biomarkers have been identified to be markedly increased in setting of IS [22–28]. Additionally, an association between more severe brain damage and vigorous inflammatory/immune responses [23, 29, 32–36] as well as lower LVEF [22, 23] has been clearly established by previous studies [22-29, 32-37]. An essential finding in the present study was that the circulating and splenic levels of inflammatory/immune reactions were found to be remarkably increased in BD animals as compared with the control animals. Our findings were comparable with the findings of the previous studies [23, 29, 32–36]. Importantly, these circulating and splenic levels of inflammatory and immune biomarkers were notably suppressed in BD animals after receiving ADMSC treatment. Our findings extended the findings of the previous studies [23, 29, 32–36].

Despite state-of-the-art in advances of pharmacomodulation and refinement of the immunosuppressant regimen for heart transplant patients, an effective method for resolving the occurrence of post-heart transplanted rejection is still lacking. Intriguingly, limited data have previously demonstrated that pre-transplant administration of MSCs can prolong the survival of allogeneic heart transplant through the generation of regulatory T cells and immune tolerance [50, 51]. A principal finding in the present study is that as compared with allograft animals without treatment, pre-heart transplantation of ADMSC transfusion preserved the integrity of donor heart architecture after transplantation. The finding of the present study was supported by previous studies [50, 51]. Another principal finding in the present study was that post-heart transplanted ADMSC therapy further protected the donor heart from acute rejection damage. The most important finding in the present study was that pre- and post-heart transplant ADMSC treatment offered a further protective effect on the donor heart. In addition to extending the results of previous studies [50, 51], our findings highlight the need for a prospective clinical trial to test the safety and efficacy of MSC therapy for prolonged heart allograft survival.

### Study limitations

This study has some limitations. First, the impact of allogenic ADMSC on protecting the remote organs (i.e., heart, liver and kidney) against the BD-induced damage and transplanted heart against acute graft versus host disease was only studied for a period of 5 days. Thus, we do not know the long-term effect of ADMSCs on protecting these organs. Second, immunosuppressant drugs such as cyclosporine or tacrolimus were not utilized in the current study. Thus, we do not know whether combined therapy with ADMSC and immunosuppressant drugs are superior to either one for protecting the transplanted heart against graft versus host disease, especially when the long-term heart transplantation outcome is taken into consideration.

In conclusion, the results of the present study demonstrated that allogenic ADMSC therapy significantly protected against BD-induced remote organ damage mainly through suppressing the inflammatory immune reactions. Additionally, allogenic AMDSC treatment reduced acute graft versus host injury in the transplanted heart, suggesting that MSCs may play an important accessory role as an immunosuppressant drug to reduce the occurrence of post-heart transplantation rejection.

## MATERIALS AND METHODS

### Ethics

All animal experimental procedures were approved by the Institute of Animal Care and Use Committee at Kaohsiung Chang Gung Memorial Hospital (Affidavit of Approval of Animal Use Protocol No. 2011053001) and performed in accordance with the Guide for the Care and Use of Laboratory Animals [The Eighth Edition of the Guide for the Care and Use of Laboratory Animals (NRC 2011)].

Animals were housed in an Association for Assessment and Accreditation of Laboratory Animal Care International (AAALAC)-approved animal facility in our hospital with controlled temperature and light cycle (24°C and 12/12 light cycle).

### Induction of brain death in the animal model (referred to Figure 1)

For induction of brain death, animals in each group were anesthetized by inhalational of 2.0% isoflurane on a warming pad at 37°C and were placed in the prone position as previously described [52] with slight modifications to mimic the clinical scenario of sudden-onset increase in intracranial pressure as in severe traumatic intra-cranial hemorrhage that results in brain-stem failure. Following an incision made on the skin covering the skull and dissection of the epicranial muscles and periosteum, a 4F angioplasty balloon catheter was carefully introduced into the supradural space inside the cranial cavity of the rat through a blur hole on the skull at the paramedian space near the frontal transverse sinus made with a dental drill. Each animal was endotracheally intubated with positive-pressure ventilation (180 mL/min) with room air using a small animal ventilator (SAR-830/A, CWE Inc., U.S.A.) at a ventilation rate of about 60/min and BD was induced by injecting 0.5 mL of distilled water through the catheter into the supradurally implanted balloon. Heart rate and blood pressure were continuously monitored during the whole procedure.

### Criteria for identification of BD in animals (referred to Figure 1)

BD manifested as an immediate and significant reduction in system arterial blood pressure (SBAP) and the power density of low-frequency (LF) components of SABP and had to include the following criteria:

1. Cessation of spontaneous respiration (i.e., complete dependence on mechanical ventilation)
2. Irreversible deep coma (i.e., lack of response and reflex to pain elicited by pinching the foot with forceps)
3. Fixed and dilated pupils without light reflex regardless of the intensity of light (i.e., fixed and dilated pupils without reflex to light)

The sum of power density during the period of the low-frequency (LF; 0.25-0.8 Hz in rat) components in the blood pressure (BP) and heart rate (HR) spectra, along with the average values and of mean BP and HR were computerized in the present study [53–55].

### Animal grouping

To determine the impact of BD on remote organ damage, pathogen-free, adult male Fischer344 (F344) rats (n=24) weighing 250-280 g (Charles River Technology, BioLASCO Taiwan Co. Ltd., Taiwan) were utilized in the present study. These animals were randomly divided into three groups: sham-control (SC), BD and BD^MSC^ [BD + allogenic ADMSC (1.2 × 10^6^ cells: derived from other additional F344 (i.e., the donors for ADMSC) by intravenous transfusion 3 h after BD procedure)].

To elucidate the protective effect of allogenic ADMSC on transplanted heart against post-transplant rejection, animals (n = 8 in each group of F344 and Lewis) were categorized into four groups: BD-T [F344 heart (i.e., donor) was transplanted into Lewis (i.e., recipient) by 6 h after BD], BD-T^MSC(D1/3)^ [BD induction in F344 for 6 h followed by F344 heart transplanted into Lewis and allogenic ADMSCs (1.2 × 10^6^ cell/each time) (i.e., ADMSCs from F344 donor) were transfused into Lewis (i.e., recipient) at days 1 and 3 after heart transplantation), BD-T^MSC(3h)^[BD + ADMSC (1.2 × 10^6^ cells) at 3 h and heart transplantation at 6 h after BD], and BD-T^MSC(3h, D1/3)^ [BD + ADMSC (1.2 × 10^6^ cells) at 3 h and heart transplantation at 6 h after BD, followed by ADMSC therapy to recipient by days 1 and 3 after transplantation procedure]. All of the animals were euthanized and the hearts were harvested by day 5 after heart transplanted procedure for individual study.

### Procedure and protocol of heart transplantation

#### Stage 1

1. After being anesthetized with 2% inhalational isoflurane, the recipient rat (i.e., Lewis) was placed in a supine position with its four limbs secured on the operation table with adhesive tape.
2. A midline skin incision was made vertically between the mandible and the sternal notch.
3. The external jugular vein was identified and dissected from the surrounding tissue until a length between 0.5 and 1.0 cm was free. The side branches of the vein were ligated and divided, while the vein was clamped proximal with a vascular clip.
4. The isolated vein was then inserted through a segment of 18G plastic venous catheter of length 1.5-2.0 mm that serves as a cuff. The portion of vein that passed through the cuff was turned inside out to cover the cuff on which a piece of 6-0 silk was used to secure the venous segment on the cuff.
5. The ipsilateral internal carotid artery was identified and dissected free from the surrounding structures until a length of 0.5 - 1.0 cm was freed. While the proximal part of the arterial segment was clamped with a vascular clip, it was ligated and divided distally.
6. The dissected artery was then inserted through a segment of 22G plastic venous catheter of length 1.5-2.0 mm that serves as a cuff. The portion of the artery that passed through the cuff was turned inside out to cover the cuff on which a piece of 6-0 silk was tied to secure the arterial segment on the cuff.

#### Stage 2

1. After being anesthetized with 2% isoflurane, a midline abdominal incision was made on the donor rat (i.e., F344) between the xyphoid process and the symphysis pubis. The infra-hepatic vena cave was identified to which 10 mL physiological saline with 10% heparin was infused. The heart was exposed by opening up the thoracic cavity with a midline incision through the sternum. After moistening the heart with 0.5 mL heparinized saline, the aorta and pulmonary arteries were dissected free from the surrounding structures and divided. The heart graft was harvested after ligation of the superior and inferior vena cava as well as the pulmonary veins.
2. The heart graft was placed on the neck of the recipient rat next to the recipient vessels to be anastomized and was kept moist with normal saline. The dissected segment of the internal carotid artery of the recipient was inserted into the aorta of the heart graft. A piece of 6-0 silk was tied over the inserted segment. Similarly, the dissected segment of the external jugular vein was inserted into the pulmonary artery of the heart graft, followed by securing the anastomosis by tying with a piece of 6-0 silk. The vascular clips on the external jugular vein and internal carotid artery of the recipient were then removed to allow perfusion and resumption of beating of the heart graft.
3. Following placement of the heart graft back to the neck, the skin of the neck was closed with 5-0 Nylon. The recipient rat was allowed to recover from anesthesia in a portable animal intensive care unit (ThermoCare) for 24 hours.

### Isolation of adipose tissue from additional 24 F344 for culturing ADMSCs

The procedure and protocol for ADMSC isolation and culturing have been described in our previous reports [42-44, 49]. Briefly, animals (i.e., additional F344) were anesthetized with inhalational 2.0% isoflurane 14 days before the BD procedure to harvest the adipose tissue surrounding the epididymis. Then 200-300 μL of sterile saline was added to every 0.5 g of adipose tissue to prevent dehydration. The tissue was then cut into < 1 mm^3^ size pieces using a pair of sharp, sterile surgical scissors. Sterile saline (37°C) was added to the homogenized adipose tissue in a ratio of 3:1 (saline: adipose tissue) by volume. Isolated allogenic ADMSCs were cultured in a 100 mm diameter dish with 10 mL DMEM culture medium containing 10% FBS for 14 days.

### Collection of blood samples and specimens of major organs for specific studies

The blood samples were collected prior to and at 6 and 48 h after BD induction to measure the CD3/CD4^+^, CD8/CD4^+^, Treg^+^ and LY6G^+^ cells by flow cytometry, and plasma levels of TNF-α, IL-β, IL-6 and MPO using ELISA assessment. Additionally, blood samples were collected from the spleen prior to euthanizing the animals for analysis of immune cells (CD3/CD4^+^, CD8/CD4^+^, Treg^+^).

### Flow cytometric quantification of helper T cells and cytotoxic T cells

The procedure and protocol of flow cytometry for identification and quantification of circulating and splenic immune cells were based on our previous report [44]. Briefly, the peripheral blood mononuclear cells (PBMCs) and splenocytes were obtained from the tail vein using a 27# needle. PBMCs and splenocytes (1.0 × 10^6^ cells) were triple-stained with FITC-anti-CD3 (BioLegend), PE-anti-CD8a (BD Bioscience), and PE-Cy™5 anti-CD4 (BD bioscience). The numbers of CD3^+^CD4^+^ helper T cells and CD3^+^CD8^+^ cytotoxic T cells were analyzed using flow cytometry (FC500, Beckman Coulter).

### Immunohistochemical (IHC) and immunofluorescent (IF) staining

The procedure and protocol of IF staining have been described in details in our previous reports [42-44, 49]. For IHC and IF staining, rehydrated paraffin sections were first treated with 3% H_2_O_2_ for 30 minutes and incubated with Immuno-Block reagent (BioSB) for 30 minutes at room temperature. Sections were then incubated with primary antibodies specifically against F4/80 (1:100, Abcam), CD14 (1:300, Bioss), CD68 (1:100, Abcam), CD69 (1:400, Gene Tex), CD3 (1:400, Abcam), CD4 (1:200, Novus Biologicals) and γ-H2AX (1:500, Abcam) while sections incubated with the use of irrelevant antibodies served as controls. Three sections of liver specimen from each rat were analyzed. For quantification, three randomly selected HPFs (200x or 400x for IHC and IF studies) were analyzed in each section. The mean number of positively-stained cells per HPF for each animal was then determined by summation of all numbers divided by 9.

### Western blot analysis

The procedure and protocol for Western blot analysis were based on our recent reports [42-44, 49]. In details, equal amounts (50 μg) of protein extracts were loaded and separated by SDS-PAGE using acrylamide gradients. After electrophoresis, the separated proteins were transferred electrophoretically to a polyvinylidene difluoride (PVDF) membrane (Amersham Biosciences). Nonspecific sites were blocked by incubation of the membrane in blocking buffer [5% nonfat dry milk in T-TBS (TBS containing 0.05% Tween 20)] overnight. The membranes were incubated with the indicated primary antibodies [cleaved poly (ADP-ribose) polymerase (PARP) (1:1000, Cell Signaling), phosphorylated (p)-Smad3 (1:1000, Cell Signaling), p-Smad1/5 (1:1000, Cell Signaling), transforming growth factor (TGF)-ß (1:500, Abcam), bone morphogenetic protein (BMP)-2 (1:500, Abcam), TNF-α (1:1000, Cell Signaling), nuclear factor (NF)-κB (1:600, Abcam), interleukin [6]-1β (1:1000, Cell Signaling), IL-6 (1:750, Abcam), high-mobility group protein-1 (HMG-1) (1:1000, Cell Signaling), IL-10 (1:1000, Abcam), IL-34 (1:400, Abcam), matrix metalloproteinase (MMP)-9 (1:2000, Abcam), macrophage inflammatory protein (MIP)-1α (1:1000, Abcam), and actin (1:10000, Millipore)], for 1 hour at room temperature. Horseradish peroxidase-conjugated anti-rabbit immunoglobulin IgG (1:2000, Cell Signaling) was used as a secondary antibody for one-hour incubation at room temperature. The washing procedure was repeated eight times within one hour. Immunoreactive bands were visualized by enhanced chemiluminescence (ECL; Amersham Biosciences) and exposed to Biomax L film (Kodak). For the purpose of quantification, ECL signals were digitized using Labwork software (UVP).
